# A Self-Adaptive Steered Molecular Dynamics Method Based on Minimization of Stretching Force Reveals the Binding Affinity of Protein–Ligand Complexes

**DOI:** 10.3390/molecules201019236

**Published:** 2015-10-22

**Authors:** Junfeng Gu, Hongxia Li, Xicheng Wang

**Affiliations:** 1 State Key Laboratory of Structural Analysis for Industrial Equipment, Department of Engineering Mechanics, Dalian University of Technology, Dalian 116023, China; E-Mail: guixum@dlut.edu.cn; 2 School of Mechanical Engineering, Dalian University of Technology, Dalian 116023, China; E-Mail: hxli@dlut.edu.cn

**Keywords:** binding affinity, steered molecular dynamics, rupture force, protein–ligand unbinding, optimization

## Abstract

Binding affinity prediction of protein–ligand complexes has attracted widespread interest. In this study, a self-adaptive steered molecular dynamics (SMD) method is proposed to reveal the binding affinity of protein–ligand complexes. The SMD method is executed through adjusting pulling direction to find an optimum trajectory of ligand dissociation, which is realized by minimizing the stretching force automatically. The SMD method is then used to simulate the dissociations of 19 common protein–ligand complexes which are derived from two homology families, and the binding free energy values are gained through experimental techniques. Results show that the proposed SMD method follows a different dissociation pathway with lower a rupture force and energy barrier when compared with the conventional SMD method, and further analysis indicates the rupture forces of the complexes in the same protein family correlate well with their binding free energy, which reveals the possibility of using the proposed SMD method to identify the active ligand.

## 1. Introduction

The calculation of binding free energies is still one of the greatest challenges of condensed-phase simulation. Of particular difficulty is the calculation of binding free energies that involves substantial reorganization of the environment, as in the case of the binding of different ligands to a protein. Predicting the binding free energy of ligands to macromolecules can be of great practical value in identifying novel molecules that can bind to target receptors and act as therapeutic drugs. During the past several decades, with the fast development of theoretical research of receptor–ligand interaction and drug molecular design methods, research into the protein–ligand binding free energy method has gained increasing attention, and many methods have been proposed. Typically, these methods can be used either to calculate the free energy of the bound and unbound states separately, in approaches such as the Molecular Mechanics Poisson-Boltzmann Surface Area (MM-PB/SA) method, the Molecular Mechanics Generalized-Born Surface Area (MM-GB/SA) and Linear Interaction Energy (LIE) method [1,2,3,4,5,6,7], or to evaluate the free energy difference between bound and unbound states, such as Free Energy Perturbation (FEP) and thermodynamic integration (TI) [8,9,10,11,12]. Recently, various types of biased sampling methods along certain reaction coordinates have also been found successful in free energy calculation of the biomolecules, including metadynamics, adaptive force bias, umbrella sampling, steered molecular dynamics (SMD), *etc.* [13,14,15,16,17].

As a complementary approach for experimental measurements, the SMD method was introduced around 1997 [18], and has been proved to be a valuable tool to reveal the details of underlying events and information about the energy landscape of receptor–ligand unbinding on the atomic level [19]. Specifically, Jarzynski equation and Crooks fluctuation theorem have indicated the potential of mean force can be obtained from non-equilibrium SMD simulations [20,21]. In SMD experiments, several pulls are simulated in one (forward) or two (forward and reverse) directions. One-directional SMD simulations combined with Jarzynski equation have been successfully used to compute binding free energy on several biomolecule systems [22,23,24,25,26], and some approaches aimed at finding important pathways to overcome the limitation of implementing the Jarzynski equation have also been proposed [27,28,29,30,31]. In addition, binding free energy calculations using bidirectional SMD simulations have appeared recently in the literature [32,33,34,35,36,37]. These researches reveal that the SMD method has the potential to reveal the binding energy of protein–ligand complexes and distinguish strong binders from weak ones.

In several recent researches, the protein–ligand rupture force obtained from SMD simulations was used as a measurement of the binding energy: the larger the rupture force of the receptor–ligand system is, the higher its binding affinity will be, and this hypothesis has been used in lead compound screening [38,39,40,41]. However, the practicality and generality of the method in revealing the binding energy of the ligand is still in need of further research. Firstly, the choice of the initial pulling direction is often randomly done or the result of guessing according to the structural information, so an inappropriate pulling direction may be determined. Even when the pulling direction is correctly chosen, the direction is fixed during the unbinding processes, which may be inconsistent with the actual situation, and an improper unbinding pathway may be obtained, or the ligand may be fail to be disassociated from the receptor. Therefore, the force profile along the improper pathway may not be able to reveal the real binding energy of the ligand.

Our group has proposed a new steered molecular dynamics strategy with pulling direction optimization. The strategy aims to more effectively determine the pathway of ligand dissociation, which has been applied on some typical protein–ligand and protein–protein complexes, and the new pathway was found to have a smaller rupture force and lower energy barrier than that of the conventional SMD [42,43,44]. In this paper, the proposed self-adaptive SMD method is attempted in order to rank the binding energy of protein–ligand complexes. During the unbinding process of the self-adaptive simulation, the pulling force along the direction is treated as the optimization objective, and the pulling direction is chosen with a specified genetic algorithm based on information entropy and multi-population techniques. Therefore, the SMD method can be used to find the most likely dissociation pathway of the receptor–ligand system, and to evaluate the binding energy of the protein–ligand complex.

In the following sections, we firstly present the self-adaptive algorithm to overcome the limitations of the conventional SMD. Then, two families of protein–ligand complexes are used to investigate the relationship between the experimental binding free energy and their rupture force from the obtained self-adaptive SMD method. The results show that the presented scheme can reveal the binding affinity difference, thus hopefully making the proposed SMD method a suitable tool to identify the active ligand.

## 2. Models and Methods

### 2.1. The Self-Adaptive SMD Strategy

The conventional SMD simulation is meant to reproduce principles of atomic force microscopy experiments in which one biomolecule is pulled by a cantilever. The center of mass of the pulled molecule will be harmonically constrained with force constant *k* to move with velocity *v* in the direction unit vector **n**. SMD thus has the following potential:

(1) $$U=\frac{1}{2}k{\left[vt-\left(R(t)-R(0)\right)\cdot n\right]}^{2}$$

In conventional SMD, the pulling direction **n** of the pulling force remains unchanged once it is assigned at the very start, and the choice of the initial pulling direction is randomly done or the result of guessing according to the structural information. However, the unchanged artificial pulling direction is likely to deviate from the natural unbinding pathway of the complex, which will lead to error estimation of the unbinding energy or even failed unbinding simulation in some cases.

According to recent researches, the unbinding rupture force correlates well with the binding energy. Therefore, the proposed method treats the pulling force as an indication of the unbinding energy barrier, and it is used as the optimization objective of the pulling direction during the unbinding simulation. The self-adaptive SMD simulation can be described as the following optimization problem:

(2) $$\begin{array}{l} \min\bar{F}\left(t,T,\varphi ,\theta \right)\\ s.t.\{\begin{array}{c} -\pi \leq \varphi \leq \pi \\ 0\leq \theta \leq \frac{\pi }{2} \end{array} \end{array}$$

where $\bar{F}(t,T,\varphi ,\theta )=-\int_{t}^{t+T}\nabla Ud\tau /T$ is the average pulling force applied to the pulled molecule during a period of time *T* (1 ps is adopted in this paper) from the time point *t* with a new pulling direction. The new pulling direction is determined by three factors: the initial direction **n**_0_, nutation angle θ and procession angle φ. The range of the nutation angle θ is between 0 and π/2 rad to ensure the pulling direction **n** in the hemispherical space along the initial direction **n**_0_, and the procession angle φ varies from -π to π. The optimization time point *t* is chosen with a minimal time interval *t*_0_ and a minimal cut-off force *f*_0_. When the simulation time period counting from the last optimization time point exceeds *t*_0_ and, meanwhile, the average pulling force during the last 1 ps is larger than *f*_0_, then the pulling direction optimization is executed at the current time point *t*.

The optimization problem is resolved with a multi-population genetic algorithm, which will be described in the next section. The optimization result, *i.e.*, the new pulling direction, will be adopted for the following SMD simulation until the next direction optimization. Therefore, the pulling direction will adjust according to the pulling force until the ligand is pulled out of the binding pocket.

### 2.2. The Multi-Population Genetic Algorithm

We use in a multi-population genetic algorithm based on information entropy to solve Equation (2) [45]. The algorithm begins with generating arbitrary *m* populations with the same search space, *i.e.*, the initial design space, and *m* adopts 16 in this paper. The search space of each population is narrowed according to the following formula:

(3) $$\begin{array}{l} E(K)=(1-p_{j})E(K-1)\\ \underline{d_{i}}(K)=\max\{\left[d_{i}^{*}(K)-0.5(1-p_{j})E(K)\right],\underline{d_{i}}(0)\}\\ \bar{d_{i}}(K)=\min\{\left[d_{i}^{*}(K)+0.5(1-p_{j})E(K)\right],\bar{d_{i}}(0)\} \end{array}$$

where *E*(*K*) is the searching space at the *K*th iteration, $\underline{d_{i}}(K)$ and $\bar{d_{i}}(K)$ are the updated lower and upper limits of the *i*th design variable respectively, and $d_{i}^{*}(K)$ is the *i*th design variable value of the best member in the *j*th population. Assuming that *F_j_*(*x*) (*j* = 1, …, *m*) stands for the best value of the fitness function in the *j*th population, the optimum problem is:

(4) $$\min\;F_{j}(x)\;j=1,\;2,\;...,\;m$$

When applying information entropy to the optimization problem, the model can be given as follows:

(5) $$\{\begin{array}{l} \min\sum_{j=1}^{m}p_{j}F_{j}(x)\\ \min H=-\sum_{j=1}^{m}p_{j}\ln p_{j}\\ s.t.\sum_{j=1}^{m}p_{j}=1,p_{j}\in \left[0,1\right] \end{array}$$

where *H* represents the information entropy, *p_j_* stands for the probability of optimal solution of Equation (4). It can be proved that Problems (4) and (5) have the same optimal solution. Problem (5) is a multi-objective problem, and it can be transformed into a single objective one with the weighted coefficient method so that it can be solved with the genetic algorithm.

### 2.3. Choice of Receptor–Ligand Complexes

**Figure 1:**
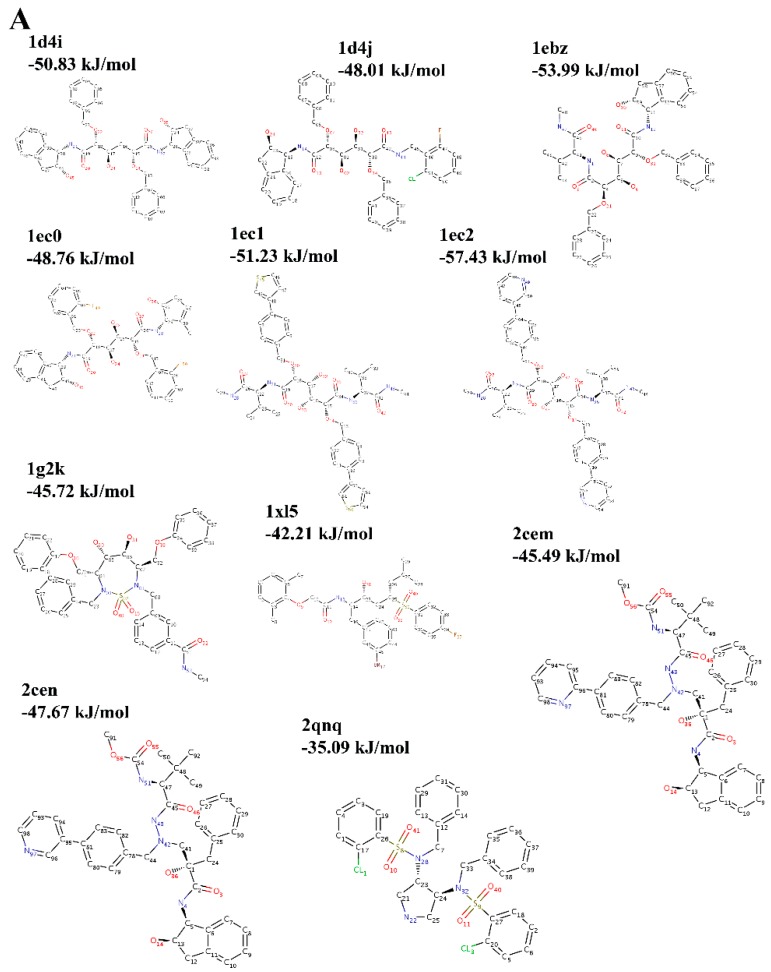

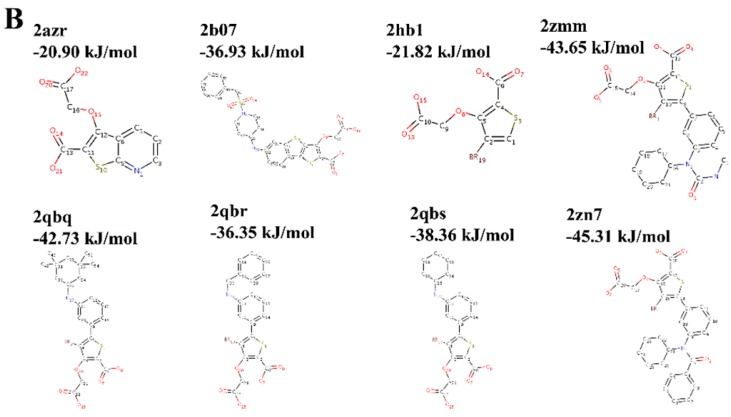
The ligand structural formulas of the complexes in the two families: (**A**) HIV-1 protease and (**B**) Tyrosine-protein phosphatase.

In order to evaluate the relationships between the rupture force obtained from the self-adaptive SMD procedure and the binding free energy of the complex, two protein families, HIV-1 protease (L63P) and tyrosine-protein phosphatase, are simulated to dissociate the ligands from their receptors. The structures and the binding free energy data are derived from the community structure-activity resource (CSAR, http://www.csardock.org), which provides experimental datasets of crystal structures and binding affinities for diverse protein–ligand complexes. The families are grouped by 100% sequence identity of the receptor protein, so we can examine relative binding trends or rankings of a series of compounds bound to the same protein. The HIV-1 protease family contains 11 complexes (2qnq, 1ebz, 1g2k, 1xl5, 1ec2, 1ec1, 1ec0, 1d4i, 1d4j, 2cen, 2cem) and the tyrosine-protein phosphatase contains eight complexes (2b07, 2zmm, 2zn7, 2hb1, 2azr, 2qbq, 2qbs, 2qbr). Figure 1 gives the ligand structural formulas of all the complexes in both families, and the binding free energy is also given in the figure. All the ligands are distinct even in the same family, and therefore bind to different binding sites.

All the simulations are performed with molecular dynamics simulation program GROMACS 3.2.1 [46], and the GROMOS96 force field is used [47,48]. Before the SMD simulation, each complex is embedded in a square periodic box, in which the shortest distance between the protein surface and the box walls is larger than 1.0 nm. The box is filled with water molecules represented by the simple point charge model [49]. At the same time, in order to balance the net charge of the system, favorable counterions such as Na+ or Cl− are added into the box, and GROMOS96 force field is also used for the counterions. The system is energy-minimized without constraints with the steepest descent method, and then 100 ps position-restrained molecular dynamics at 300 K and 1.0 bar is performed to make sure the equilibration of the solvent molecules and ligands with the protein is maintained. In this run, the atom positions of the protein are restrained to restrict their movement in the simulation. Next, 100 ps MD simulation without position-restraint is performed to ensure the equilibration of the system. At last, the ligand is pulled with an external force in NPT ensemble at 1.0 bar and 300 K, whose direction will adjust automatically with the proposed method, and simulations are performed with 2 fs time steps. During the simulation, the translation of the center of the mass of the protein is removed at every step. The initial structures of the SMD simulations are derived from the equilibration stage. For both families, the initial pulling direction is along a line which is determined by the locations of two atoms from the secondary structures, which form the skeleton of the protein and are relatively stable to ensure the consistency of the pulling direction in the same family. The initial direction will make sure the ligand is pulled against the protein and toward the solvent, and following this direction will ensure as little contact as possible with atoms of the protein. The force constant of the spring is 200 kJ·mol^−1^·nm^−2^, and the rate at which the spring is pulled is 0.002 nm·ps^−1^. When the minimum distance between the atoms of the receptor and the ligand reaches a cut-off value of 0.6 nm, the corresponding time is taken as the ending time of the dissociation simulation.

## 3. Results and Discussion

### 3.1. The Influence of the Cut-Off Force

**Figure 2:**
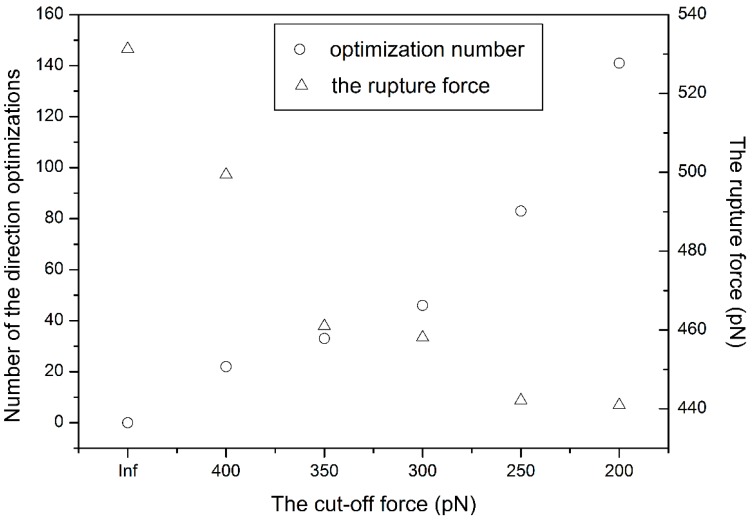
The influence of the cut-off force on the rupture force of 1d4i.

**Figure 3:**
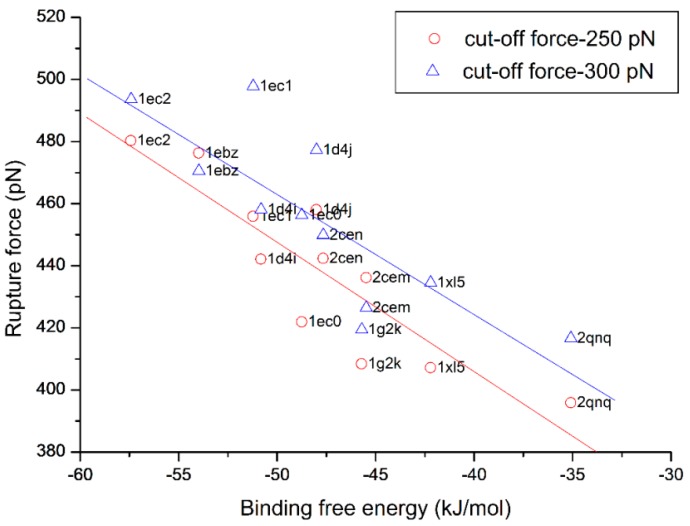
The influence of the cut-off force on the rupture force of the complexes in the HIV-1 protease family.

In the proposed self-adaptive SMD method (SA-SMD), two parameters, *t*_0_ and *f*_0_, are adopted to control the optimization process, because it is impossible to execute direction optimization on all the time points, which is not necessary and impractical as the computation time is too long. Considering the efficacy of the proposed method, *t*_0_ is set as 5 ps in this paper. *f*_0_ is a minimal cut-off force for the direction optimization, which will have an notable influence on the rupture force. For studying the relationship between the cut-off force and the rupture force, one simulation experiments is firstly made on the 1d4i complex with conventional SMD method (C-SMD), *i.e.*, no direction optimizations are executed in the simulation, and the rupture force is 531.3 pN. The rupture force corresponds to the maximum time-averaged force encountered while pulling the ligand out of the protein during simulation, where averages are taken over 1 ps time intervals. Then, five other simulations are made independently with the SA-SMD method. All five simulations adopt the same simulation parameters as described in Section 2 except the cut-off force, which is set as 400, 350, 300, 250, 200 pN, respectively. Number of the direction optimizations and the rupture force of each simulation are extracted and plotted in Figure 2, and the Inf label of cut-off force in the figure indicates the simulation is executed with C-SMD. As shown in the figure, when the cut-off force decreases, the number of the direction optimizations improves, which means a lower cut-off force will improve the chances for direction optimization. Meanwhile, the rupture force will be reduced when lower cut-off force is adopted, but the rate of the reduction gradually slows down. As indicated in the figure, when the cut-off forces are 250 and 200, their optimization numbers are very different, but the rupture forces are almost equivalent. In addition, simulations to study the influence of the cut-off force to rupture force are made on the HIV-1 protease family. Each complex in the family is simulated independently using SA-SMD with two cut-off forces: 250 and 300 pN. The rupture force *vs.* its binding free energy derived from experiment is plotted in Figure 3. As shown in the figure, the averaged rupture force obtained with 300 pN cut-off force is slightly larger than with the 250 pN cut-off force, which is consistent with Figure 2. Correlation analysis is also performed on these two sets of dots in Figure 3, and the linear relationship is observed in both sets. The correlation coefficients for 250 and 300 pN cut-off forces are 88.4% and 81.1%, respectively. Therefore, considering both the simulation efficacy and the optimization effect, 250 pN is adopted as the cut-off force in all of the following simulations.

### 3.2. The Influence of the Pulling Rate

**Figure 4:**
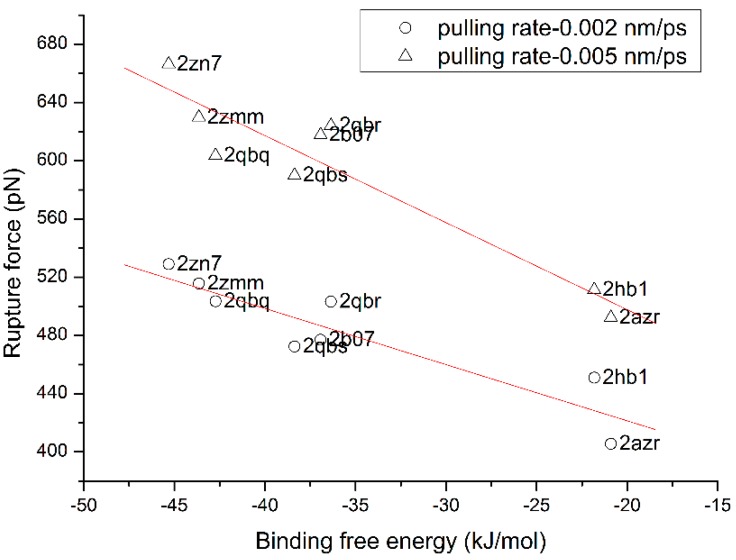
The influence of the pulling rate on the rupture force of the complexes in the Tyrosine-protein phosphatase family.

For identifying the influence of the pulling rate on the rupture force, another simulation experiment is made on the tyrosine-protein phosphatase family. Each complex in the family is simulated independently using SA-SMD with two pulling rates: 0.002 and 0.005 nm/ps. The rupture force *vs.* its binding free energy which is obtained from experiment is plotted in Figure 4. As shown in the figure, the rupture forces of the complexes in the family with 0.005 nm/ps pulling rate are obviously larger than with the pulling rate of 0.002 nm/ps. In addition, with both pulling rates, the rupture force shows a linear relationship with the binding free energy, though the slopes of the fitted lines are different.

### 3.3. The Optimized Unbinding Process

**Figure 5:**
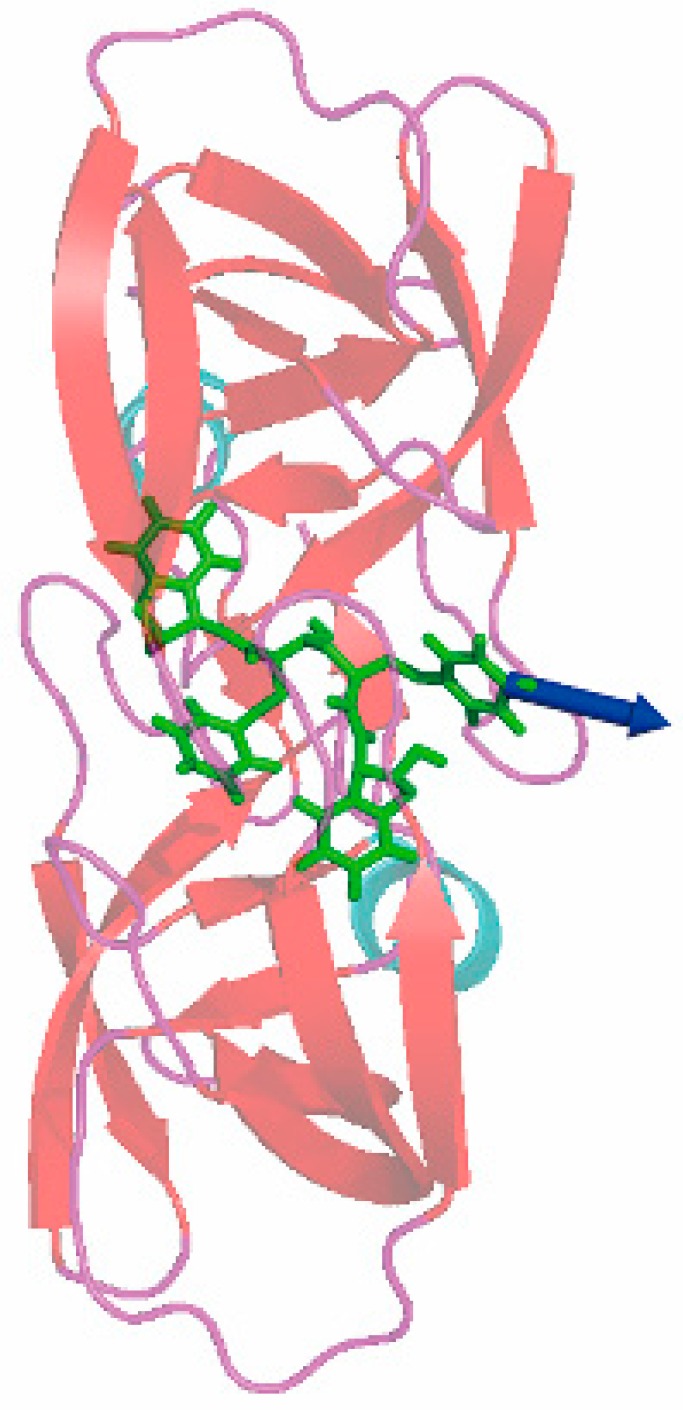
The initial structure of the steered molecular dynamics simulation of 1d4i, and the initial pulling direction is labeled with a blue arrow.

**Figure 6:**
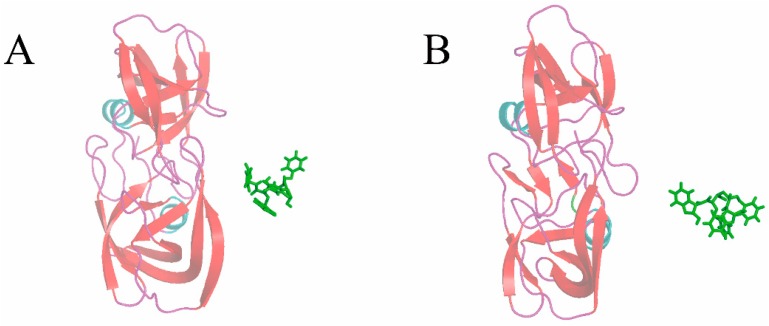
Final structures of the steered molecular dynamics simulations of 1d4i with (**A**) C-SMD and (**B**) SA-SMD methods.

**Figure 7:**
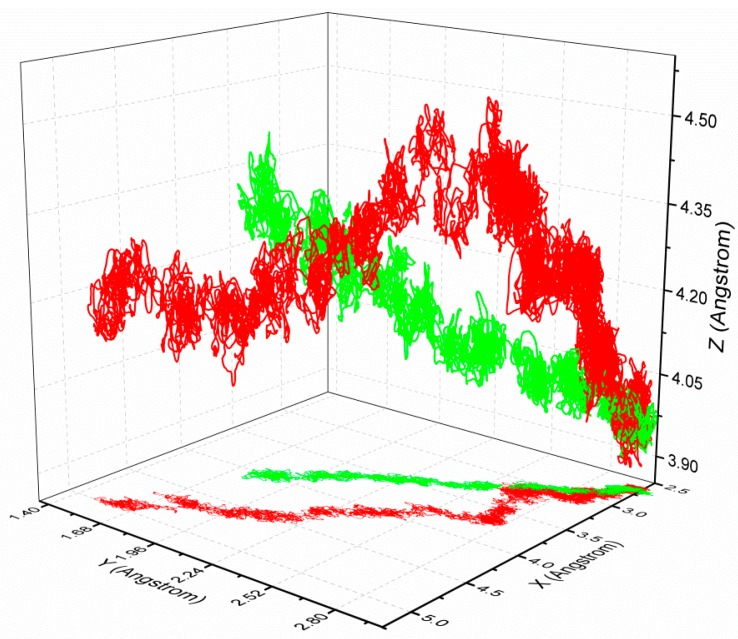
Ligand trajectories of 1d4i with C-SMD (shown in green line) and SA-SMD (shown in red line).

In this section, the 1d4i complex from the HIV-1 protease family is taken as an example to expound the protein–ligand unbinding process during simulation with SA-SMD. The simulation results of 1d4i with C-SMD are also given for comparison. Figure 5 gives the initial structure of the SMD simulations, and the initial pulling direction is also given with a blue arrow. The end time of the C-SMD and SA-SMD simulations is 950 ps and 1960 ps, respectively, and Figure 6 gives the structures at the end time. The position of center of mass of the ligand is recorded during the simulation, and the ligand trajectories derived from SA-SMD and C-SMD methods are shown in Figure 7 (cannot find), which reveals two distinct dissociation pathways of the ligand. The trajectory direction of C-SMD is monotonic, which is consistent with the initial pulling direction. Referenced to the C-SMD, the trajectory of the SA-SMD is more complicated, and several significant turning points of ligand movement direction can be observed, which leads to a more tortuous dissociation pathway and a longer dissociation time.

**Figure 8:**
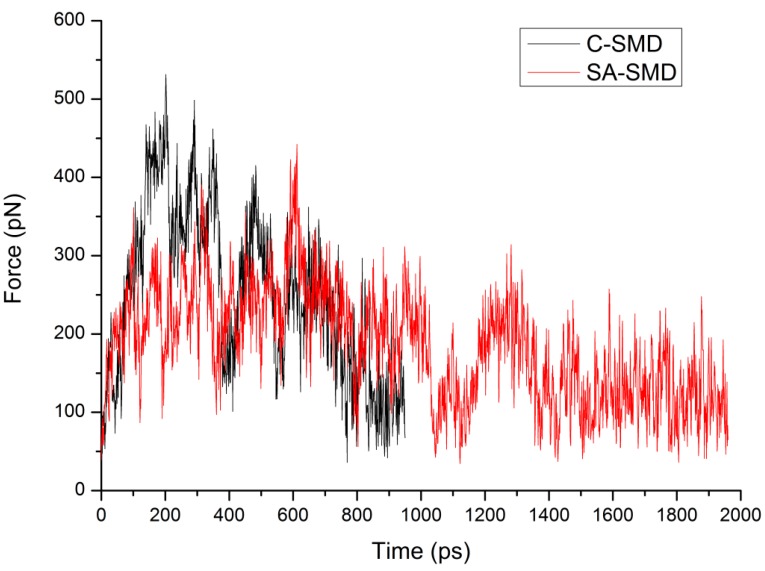
The time-averaged force profiles during the unbinding simulation of the 1d4i complex with C-SMD (shown in black line) and SA-SMD (shown in red line).

The time-averaged force profiles during the unbinding simulation of the 1d4i complex are shown in Figure 8. For the C-SMD (the black line), a steady increase of the applied force can be observed during the first 200 ps, and then it reaches the maximum force at 204 ps, which corresponds to the rupture force of the ligand unbinding along this dissociation pathway. After some fluctuation, the force value undergoes a fast decrease until the end time. For the SA-SMD simulation (the red line), a peak value can be found at 611 ps, and the force value tends to be stable after 1300 ps. It is obvious that the rupture force of the proposed SA-SMD method (442 pN) is lower than the C-SMD (531 pN). The smaller rupture force reflects the easier unbinding of the 1d4i complex with the SA-SMD method.

**Figure 9:**
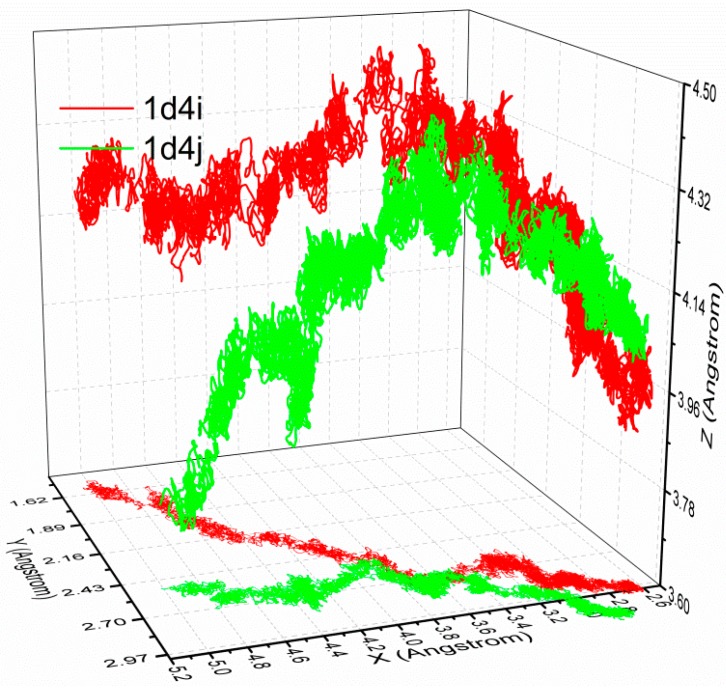
Ligand trajectories of 1d4i (shown in red line) and 1d4j (shown in green line) simulated with SA-SMD.

Then, the dissociation pathways of similar ligands in the same family are also investigated. The configurations and binding free energy of complexes 1d4i and 1d4j of the HIV-1 protease family are similar, and the trajectories of the center of mass of their ligands are plotted in Figure 9. The initial position of the ligands are (2.67, 2.97, 3.97) and (2.84, 3.14, 4.10), respectively, and the slight difference is due to separate binding and relaxation of different ligands. At the initial phase of the dissociation, the ligands are dissociated along the approximate pathways as shown in Figure 9, but different pathways are adopted in the following simulations. The results show that the dissociations with a minimum stretching force will follow different pathways even with similar ligands, which reveal the limitation of C-SMD from the other side.

### 3.4. The Relationship of the Rupture Force and Binding Free Energy

**Figure 10:**
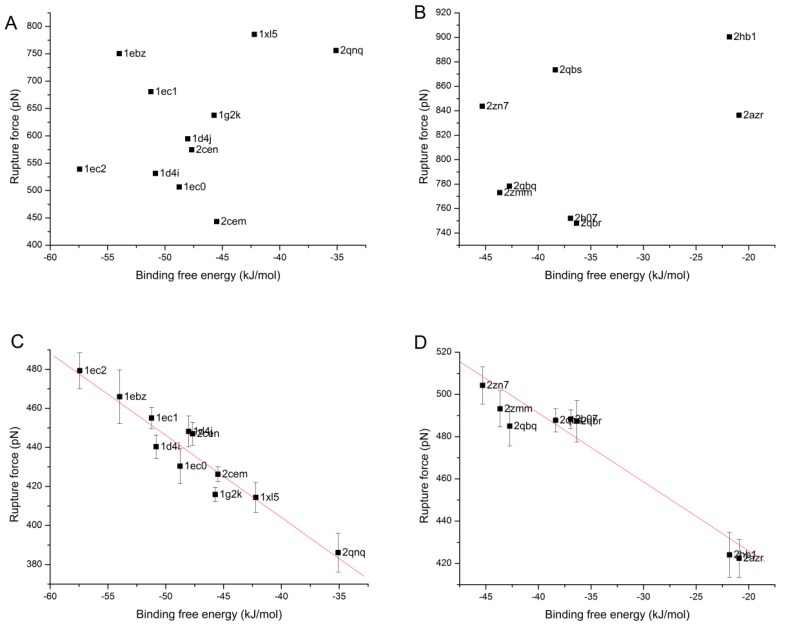
The relationship between the binding free energy and the rupture force obtained with C-SMD and SA-SMD of the complexes in the two protein families: (**A**) HIV-1 protease with C-SMD; (**B**) Tyrosine-protein phosphatase with C-SMD; (**C**) HIV-1 protease with SA-SMD and (**D**) Tyrosine-protein phosphatase with SA-SMD.

In this section, the relationship between the rupture force and the binding free energy is further investigated. Firstly, C-SMD simulation is carried out once for each complex in both families, and the rupture force of the simulation is taken and plotted in Figure 10A,B against its binding free energy. The distributions of the dots in both families are random, no inherent order is found between the rupture force got from C-SMD and binding free energy. Then, four independent simulations with SA-SMD are also carried out for each complex, and the mean value and standard error of the rupture forces of all the four simulations are calculated and plotted in Figure 10C,D. Comparison between Figure 10A–D shows that the rupture force of each complex is reduced with SA-SMD. More importantly, linear correlations are clearly observed between the rupture force and binding free energy in Figure 10C,D. The correlation factor of the HIV-1 protease family is 95.5%, and that of the Tyrosine-protein phosphatase family is 94.8%. This results show that the rupture force gained with the SA-SMD method correlates well with the experimental binding free energy.

It is noteworthy that the slopes of the fitted lines of the two protein families are inconsistent, which makes direct comparison of the binding affinity between different families unfeasible. The reason behind the phenomenon is interesting and needs to be further examined. As shown in Figure 4, the pulling rate partly determines the absolute value of the rupture force and the slope of the fitted line, and the same situation may also occur with other simulation parameters. Therefore, we thought one reason may be that the same set of simulation parameter is not adequate for different protein families, and finding the internal relationship will be the focus of our future work. In addition, another reason may be the incomplete direction optimization, because the optimization cannot be executed at all time points, which may lead to deviation from the real dissociation pathway.

This work is inspired by some exploratory researches which are based on the hypothesis that the larger the rupture force of the receptor–ligand system is, the higher its binding affinity will be, and the results from the research give support to the hypothesis. Experimental research with atomic force microscopy also indicated a remarkable correlation of the unbinding forces to the off-rates *k*_off_, and *k*_off_ usually correlates well with the equilibrium constant *K*_D_ for related receptor–ligand systems, *k*_on_ normally being confined to a rather narrow range of values. Even so, the logic and rationality of the hypothesis need to be further investigated, and there still lacks a sound theoretical background to explain the correlation between the rupture force and binding energy. Further understanding about the binding and unbinding mechanism of the complexes in the future will help to interpret the correlation, and this is another direction of our future work which will also be helpful for improving SA-SMD. Another limitation of SA-SMD is that it is impossible to execute direction optimization on all the time points, which may lead to fluctuation in the pathway choice. In this work, a relatively small optimization time interval is adopted to ensure a limited influence on the determination of the optimal dissociation pathway.

## 4. Conclusions

To sum up, this study proposed a novel SA-SMD method which can adjust the pulling direction based on optimization of the pulling force, and this method is used to discriminate the binding affinity of complexes within two families. Results show that the rupture force obtained with SA-SMD correlates well with the binding free energy in the same protein family.

This study highlights the possibility of using SMD simulation to reveal the binding affinity of a complex. With a further understanding of the binding mechanism of complexes in the same or different families, we may extend the proposed strategy for wider applications.

## References

[1] Aqvist J., Medina C., Samuelsson J.E. (1994). A new method for predicting binding affinity in computer-aided drug design. Protein Eng..

[2] Kollman P.A., Massova I., Reyes C., Kuhn B., Huo S., Chong L., Lee M., Lee T., Duan Y., Wang W. (2000). Calculating structures and free energies of complex molecules: Combining molecular mechanics and continuum models. Acc. Chem. Res..

[3] Kuhn B., Kollman P.A. (2000). Binding of a diverse set of ligands to avidin and streptavidin: An accurate quantitative prediction of their relative affinities by a combination of molecular mechanics and continuum solvent models. J. Med. Chem..

[4] Schwarzl S.M., Tschopp T.B., Smith J.C., Fischer S. (2002). Can the calculation of ligand binding free energies be improved with continuum solvent electrostatics and an ideal-gas entropy correction?. J. Comput. Chem..

[5] Homeyer N., Gohlke H. (2012). Free energy calculations by the molecular mechanics poisson-boltzmann surface area method. Mol. Inf..

[6] Wright D.W., Hall B.A., Kenway O.A., Jha S., Coveney P.V. (2014). Computing clinically relevant binding free energies of hiv-1 protease inhibitors. J. Chem. Theory Comput..

[7] Wan S., Knapp B., Wright D.W., Deane C.M., Coveney P.V. (2015). Rapid, precise, and reproducible prediction of peptide-mhc binding affinities from molecular dynamics that correlate well with experiment. J. Chem. Theory Comput..

[8] Straatsma T.P., McCammon J.A. (1991). Multiconfiguration thermodynamic integration. J. Chem. Phys..

[9] Jiang W., Hodoscek M., Roux B. (2009). Computation of absolute hydration and binding free energy with free energy perturbation distributed replica-exchange molecular dynamics. J. Chem. Theory Comput..

[10] Khavrutskii I.V., Wallqvist A. (2011). Improved binding free energy predictions from single-reference thermodynamic integration augmented with hamiltonian replica exchange. J. Chem. Theory Comput..

[11] Wang L., Wu Y.J., Deng Y.Q., Kim B., Pierce L., Krilov G., Lupyan D., Robinson S., Dahlgren M.K., Greenwood J. (2015). Accurate and reliable prediction of relative ligand binding potency in prospective drug discovery by way of a modern free-energy calculation protocol and force field. J. Am. Chem. Soc..

[12] Kaus J.W. (2015). How to deal with multiple binding poses in alchemical relative protein–ligand binding free energy calculations. J. Chem. Theory Comput..

[13] Doudou S., Burton N.A., Henchman R.H. (2009). Standard free energy of binding from a one-dimensional potential of mean force. J. Chem. Theory Comput..

[14] Buch I., Sadiq S.K., Fabritiis G.D. (2011). Optimized potential of mean force calculations for standard binding free energies. J. Chem. Theory Comput..

[15] Miao Y., Feher V.A., McCammon J.A. (2015). Gaussian accelerated molecular dynamics: Unconstrained enhanced sampling and free energy calculation. J. Chem. Theory Comput..

[16] Takahashi R., Gil V.A., Guallar V. (2013). Monte carlo free ligand diffusion with markov state model analysis and absolute binding free energy calculations. J. Chem. Theory Comput..

[17] Chen L.Y. (2015). Hybrid steered molecular dynamics approach to computing absolute binding free energy of ligand-protein complexes: A brute force approach that is fast and accurate. J. Chem. Theory Comput..

[18] Isralewitz B., Izrailev S., Schulten K. (1997). Binding pathway of retinal to bacterio-opsin: A prediction by molecular dynamics simulations. Biophys. J..

[19] Sotomayor M., Schulten K. (2007). Single-molecule experiments *in vitro* and *in silico*. Science.

[20] Jarzynski C. (1997). Nonequilibrium equality for free energy differences. Phys. Rev. Lett..

[21] Crooks G.E. (1998). Nonequilibrium measurements of free energy differences for microscopically reversible markovian systems. J. Stat. Phys..

[22] Vashisth H., Abrams C.F. (2008). Ligand escape pathways and (un)binding free energy calculations for the hexameric insulin-phenol complex. Biophys. J..

[23] Ytreberg F.M. (2009). Absolute fkbp binding affinities obtained via nonequilibrium unbinding simulations. J. Chem. Phys..

[24] Shen J., Li W., Liu G., Tang Y., Jiang H. (2009). Computational insights into the mechanism of ligand unbinding and selectivity of estrogen receptors. J. Phys. Chem. B.

[25] Zhang D., Gullingsrud J., McCammon J.A. (2006). Potentials of mean force for acetylcholine unbinding from the alpha7 nicotinic acetylcholine receptor ligand binding domain. J. Am. Chem. Soc..

[26] Nicolini P., Frezzato D., Gellini C., Bizzarri M., Chelli R. (2013). Toward quantitative estimates of binding affinities for protein–lignad systems involving large inhibitor compounds: A steered molecular dynamics simulation route. J. Comput. Chem..

[27] Schmiedl T., Seifert U. (2007). Optimal finite-time processes in stochastic thermodynamics. Phys. Rev. Lett..

[28] Vaikuntanathan S., Jarzynski C. (2008). Escorted free energy simulations: Improving convergence by reducing dissipation. Phys. Rev. Lett..

[29] Ozer G., Keyes T., Quirk S., Hernandez R. (2014). Multiple branched adaptive steered molecular dynamics. J. Chem. Phys..

[30] Giovannelli E., Gellini C., Pietraperzia G., Cardini G., Chelli R. (2014). Combining path-breaking with bidirectional nonequilibrium simulations to improve efficiency in free energy calculations. J. Chem. Phys..

[31] Chelli R., Gellini C., Pietraperzia G., Giovannelli E., Cardini G. (2013). Path-breaking schemes for nonequilibrium free energy calculations. J. Chem. Phys..

[32] Shirts M.R., Pande V.S. (2005). Comparison of efficiency and bias of free energies computed by exponential averaging, the bennett acceptance ratio, and thermodynamic integration. J. Chem. Phys..

[33] Chelli R., Procacci P. (2009). A potential of mean force estimator based on nonequilibrium work exponential averages. Phys. Chem. Chem. Phys..

[34] Nicolini P., Procacci P., Chelli R. (2010). Hummer and szabo-like potential of mean force estimator for bidirectional nonequilibrium pulling experiments/simulations. J. Phys. Chem. B.

[35] Chelli R., Marsili S., Procacci P. (2008). Calculation of the potential of mean force from nonequilibrium measurements via maximum likelihood estimators. Phys. Rev. E.

[36] Minh D.D.L., Adib A.B. (2008). Optimized free energies from bidirectional single-molecule force spectroscopy. Phys. Rev. Lett..

[37] Shirts M.R., Bair E., Hooker G., Pande V.S. (2003). Equilibrium free energies from nonequilibrium measurements using maximum-likelihood methods. Phys. Rev. Lett..

[38] Jorgensen W.L. (2010). Drug discovery: Pulled from a protein’s embrace. Nature.

[39] Colizzi F., Perozzo R., Scapozza L., Recanatini M., Cavalli A. (2010). Single-molecule pulling simulations can discern active from inactive enzyme inhibitors. J. Am. Chem. Soc..

[40] Mai B.K., Viet M.H., Li M.S. (2010). Top leads for swine influenza a/h1n1 virus revealed by steered molecular dynamics approach. J. Chem. Inf. Model..

[41] Mai B.K., Li M.S. (2011). Neuraminidase inhibitor r-125489—A promising drug for treating influenza virus: Steered molecular dynamics approach. Biochem. Biophys. Res. Commun..

[42] Liu X., Wang X., Jiang H. (2008). A steered molecular dynamics method with direction optimization and its applications on ligand molecule dissociation. J. Biochem. Biophys. Methods.

[43] Yang K., Liu X., Wang X., Jiang H. (2009). A steered molecular dynamics method with adaptive direction adjustments. Biochem. Biophys. Res. Commun..

[44] Gu J., Wang X., Yang Y. A steered molecular dynamics method for receptor-ligand unbinding based on genetic algorithm. Proceedings of the 2013 International Conference on Information Science and Cloud Computing Companion.

[45] Kang L., Li H., Jiang H., Wang X. (2009). An improved adaptive genetic algorithm for protein–ligand docking. J. Comput. Aided Mol. Des..

[46] Van Der Spoel D., Lindahl E., Hess B., Groenhof G., Mark A.E., Berendsen H.J.C. (2005). Gromacs: Fast, flexible, and free. J. Comput. Chem..

[47] Van Buuren A.R., Marrink S.J., Berendsen H.J.C. (1993). A molecular dynamics study of the decane/water interface. J. Phys. Chem..

[48] Mark A.E., van Helden S.P., Smith P.E., Janssen L.H.M., van Gunsteren W.F. (1994). Convergence properties of free energy calculations: .Alpha.-cyclodextrin complexes as a case study. J. Am. Chem. Soc..

[49] Berendsen H.J., Postma J.P.M., van Gunsteren W.F., Hermans J., Pullman B. (1981). Interaction models for water in relation to protein hydration. Intermolecular Forces.

